# Training School Teachers to Deliver a Mindfulness Program: Exploring Scalability, Acceptability, Effectiveness, and Cost-effectiveness

**DOI:** 10.1177/2164956120964738

**Published:** 2020-12-15

**Authors:** Catherine Crane, PhD, Poushali Ganguli, PhD, Susan Ball, MSc, Laura Taylor, PhD, Sarah-Jayne Blakemore, PhD, Sarah Byford, PhD, Tim Dalgleish, PhD, Tamsin Ford, PhD, Mark Greenberg, PhD, Willem Kuyken, PhD, Liz Lord, MA, Jesus Montero-Marin, PhD, Anna Sonley, MEd, Obioha C Ukoumunne, PhD, J Mark G Williams, PhD

**Affiliations:** 1 Department of Psychiatry, University of Oxford, Warneford Hospital, Oxford, UK; 2 King’s Health Economics, Institute of Psychiatry, Psychology and Neuroscience at King’s College London, London, UK; 3 NIHR CLAHRC South West Peninsula (PenCLAHRC), University of Exeter Medical School, Exeter, UK; 4 Institute of Cognitive Neuroscience, University College London, London, UK; 5 Cognition and Brain Sciences Unit, University of Cambridge, Cambridge, UK; 6 Cambridgeshire and Peterborough NHS Foundation Trust, Cambridge, UK; 7 St Luke's Campus, University of Exeter Medical School, Exeter, UK; 8 Edna Bennett Pierce Prevention Research Center, Pennsylvania State University, University Park, Pennsylvania **Current address:** Tamsin Ford is now with the Department of Psychiatry, University of Cambridge, Cambridge, UK.

**Keywords:** mindfulness-based programs, teaching competency, effectiveness, acceptability, implementation and dissemination, training

## Abstract

**Background:**

There is growing research support for the use of mindfulness training (MT) in schools, but almost no high-quality evidence about different training models for people wishing to teach mindfulness in this setting. Effective dissemination of MT relies on the development of scalable training routes.

**Objective:**

To compare 4 training routes for school teachers wishing to deliver MT differing in intensity and potential scalability, considering teaching competency, training acceptability, and cost-effectiveness.

**Methods:**

Schools were randomized to an existing route comprising an 8-session instructor-led personal mindfulness course, combined with 4-day MT program training, or 1 of 3 more scalable, lower intensity, alternatives: an instructor-led personal mindfulness course combined with 1-day MT program training, a self-taught personal mindfulness course (delivered through a course book) combined with 4-day MT program training, and a self-taught personal mindfulness course combined with 1-day MT program training.

**Results:**

Attrition from training was substantial across all routes. The instructor-led course was more effective than the self-taught course in increasing teachers’ personal mindfulness skills. Even the most intensive (existing) training route brought only 29% of the teachers *commencing* training, and 56% of those *completing* the study protocol, to the required minimum competency threshold (an advanced beginner rating on an adapted version of the Mindfulness-based Interventions Teaching Assessment Criteria). The differences in levels of competency achieved by existing training compared with the more scalable alternatives were modest, with economic evaluation suggesting that the existing route was both more expensive and more effective than lower intensity alternatives, but with no statistically significant differences between routes.

**Conclusions:**

This research questions the move toward abbreviating teacher training to increase scalability and suggests instead that many teachers require additional support to ensure competency from first delivery of MT in the classroom.

There is increasing interest in the potential benefits of introducing mindfulness training (MT) into schools to support children and young people,^1^ motivated by early findings that such training is feasible, acceptable and provides skills that promote young people’s self-regulation, mental health, and well-being.^2^ MT is one example of a broader range of social and emotional learning programs delivered in schools. Such programs intend to improve well-being, mental health, and/or social and emotional competencies. Recent meta-analytic reviews suggest potential benefits of MT on social–emotional, cognitive and behavioral functioning in children and young people, ^3,4^ with identification of small positive effects of MT on mental health outcomes when compared with a range of active control conditions (most frequently health education programs, but also yoga, a cognitive behavioral program, and a social responsibility program) in robust randomized trial designs.^5^

Despite this rationale and promising evidence base, MT, such as other psychological interventions, is inherently difficult to scale up and make widely available to all those who might benefit.^6^ There are at least 3 major challenges to scalability of MT in schools. First, schools are complex organizations and there is a relative lack of knowledge regarding the barriers and facilitators to effective implementation of MT programs. Second, scalability of MT is limited by the training demands associated with its delivery, both in terms of time commitment and financial cost of training teachers to deliver the program to their pupils. Third, the lack of evidence or consensus concerning how best to train individuals (teachers or other providers) to deliver MT contributes to uncertainty regarding how MT training might be delivered with quality and efficiency.

To begin to address these issues, research has recently examined facilitators and barriers to implementation of MT in schools^7^ and health-care services.^8,9^ These studies show that implementing MT within health and education sectors is a journey that requires both bottom-up grass roots efforts (people who act as champions, and “will go the extra mile”) and top-down facilitation (eg, investment of time and financial resources required to train teachers to deliver MT). In schools, implementation of social–emotional teaching generally and MT specifically relies on training enough teachers who can teach these programs. However, limited resources and high staff turnover currently experienced by many state-funded schools internationally^10–12^ are likely to pose significant challenges to schools’ ability to develop and maintain provision of MT over time.

There are consensus statements about how to prepare people to deliver MT.^13^ Typically, this involves first learning mindfulness personally and then going on to learn how to deliver MT to others. Because school MT curricula are typically more standardized, the training requirements for people wishing to teach MT in schools are substantially lower than for people wishing to teach MT to adults in other contexts.^14^ Nevertheless, in their current form, the demands of MT teacher training programs in schools are greater and more expensive than for many other teacher trainings. This can act as a barrier to implementation.^7^

The .b program of the Mindfulness in Schools Project (MISP)^15^ is an example of a universal school MT program, and one of the more established MT programs.^16^ It is a highly structured, manualized program, supported by PowerPoint presentations, animations, worksheets, and online resources, which is intended to provide pupils a psychoeducational context for, and experiential practice of, MT. The training route for this program follows standard MT training for teachers more broadly, comprising attendance at a personal mindfulness course followed by training to deliver the MT program to young people. However, because the .b program is more structured and manualized, the training is shorter. At the time, this study was undertaken the training route consisted of 2 components. The first, an 8-session instructor-led personal mindfulness course was intended to develop teachers’ own mindfulness skills, consistent with evidence suggesting that personal mindfulness courses have the potential to enhance teacher well-being and support teachers’ classroom teaching.^17–19^ Following a period of personal mindfulness practice, the second phase was completion of a 4-day program-based training course, designed to prepare teachers to deliver the program to their pupils.

One way to increase scalability of MT teacher training would be to replace the instructor-led personal mindfulness course with self-taught mindfulness, delivered by a course book, or over the internet. Such delivery has significant potential to increase access to personal mindfulness courses while reducing cost.^6^Self-taught mindfulness courses have already been demonstrated to improve mental health and well-being outcomes.^20^ However, some studies have identified high rates of attrition from such courses^21^ as well as small effect sizes on mindfulness and well-being outcomes.^22^

A second way to make .b MT teacher training more scalable would be to reduce the duration of the 4-day program training. The various mindfulness-based programs for teachers and students take different approaches. For example, Cultivating Awareness and Resilience in Education,^17^ a training program only for teachers, involved 30 hours training over 5 days, alongside a series of coaching calls, when tested in a trial setting. In contrast, the Learning to Breathe program, designed for delivery to students and modeled on Mindfulness-Based Stress Reduction,^23^ makes course materials freely available, recommends that teachers have their own mindfulness practice, and offers optional 2- to 3-day training workshops and supervision. Many other teacher trainings in social–emotional curricula are either self-taught or offered through workshops of varying duration. The 4-day training currently offered by MISP for teachers might plausibly be substantially reduced, to bring it more in line with other forms of in-service training and professional development offered to teachers in schools. However, whether such changes influence teacher competency is unknown.

Despite widespread consensus that effective MT within schools is likely to depend on the establishment of feasible, effective, and scalable training routes for teachers,^24^ to the best of our knowledge there is an almost complete absence of any high-quality evidence about how to deliver such training. Research has simply not addressed either the effectiveness of existing MT training for teachers or what the impact of efforts to increase scalability of training on key outcomes might be. The study reported here was designed to explore whether current opinion about best practice is in fact effective in producing teachers who can teach a standardized mindfulness program with at least adequate minimum competency and whether more scalable models would suffice. We used the current standard training pathway for school teachers wishing to teach the .b mindfulness program developed by the MISP (8 week instructor-led personal MT, followed by 4-day program training), which in the absence of any evidence, is considered the benchmark. However, to explore scalability we included 3 further routes, each of which was intended to make the training more feasible and scalable: (a) instructor-led personal MT combined with 1-day program training, (b) self-taught personal MT (delivered through a course book) combined with 4-day program training, and (c) self-taught personal MT combined with 1-day program training.

This exploratory study was intended to provide important information about the relative costs and benefits of 4 different routes for preparing teachers to deliver MT in their classrooms. Our primary outcome of interest was whether teachers would be able to teach the mindfulness program with at least adequate minimum competency on completion of training as well as the extent to which each training route improved their personal mindfulness skills. To inform this question we also considered additional outcomes of acceptability and costs of each training route. As well as answering questions about the development of scalable training routes for people preparing to teach school mindfulness programs, the study also informed an ongoing large-scale randomized trial of MT in schools.^25^

## Method

### Study Registration, Ethical Approval, and Safety

The study was registered prior to obtaining participant consent to randomization, ISRCTN18013311 (24/11/2015). http://www.isrctn.com/ISRCTN18013311. The study was reviewed and approved by the University of Oxford Medical Sciences Interdivisional Research Ethics Committee (20/03/2015, ref: MS-IDREC-C1-2015-048) and overseen by a Data Monitoring and Ethics Committee (DMEC). One participant randomized to the self-taught course reported the recurrence of a preexisting psychological disorder on a study questionnaire, which was reviewed by the DMEC and considered to be a potential adverse reaction to the personal mindfulness course.

### Design

This was a feasibility study, using a cluster randomized controlled trial (RCT) design. Participants were teachers within English secondary schools. Schools were randomized to 1 of 4 teacher training routes: instructor-led personal mindfulness course combined with 4-day MT program training, instructor-led personal mindfulness course combined with 1-day MT program training, self-taught personal mindfulness course combined with 4-day MT program training, and self-taught personal mindfulness course combined with 1-day program training. The primary outcome for the study was teachers’ competency on completion of training.

### Recruitment

Recruitment was conducted through emails sent directly to *all* secondary school head teachers and local education authorities in England, identified through a freedom of information request. In parallel, potential participant teachers and head teachers were contacted directly through professional events (eg, local head teacher meetings), and advertisement on a national online teacher forum and word of mouth. Interested individuals (whether head teachers or teaching staff) were invited to contact the research team.

Schools were eligible for inclusion if they were willing to release participating staff for training and to subsequently timetable each participating teacher to deliver the 10-week MT program to at least one class of pupils during regular school hours. Schools were ineligible if they were currently receiving external intervention for poor standards; had only an interim head teacher in post; had delivered an MT program to their pupils as part of their general provision in the previous 12 months; were located in a region that was so geographically remote that an appropriately trained mindfulness instructor could not be identified to deliver the personal MT to participant teachers within the school, or finally; were unable to identify 3 or more participating teachers.

In eligible schools, there were also inclusion requirements for teachers. Participating teachers were eligible if they held qualified teacher status or if unqualified had at least 5 years teaching experience; were willing and able to undertake personal MT and to deliver the MT program to their pupils; and provided informed consent. Teachers were ineligible if they were planning to leave the teaching profession within the next 12 to 18 months; were on a temporary contract; had completed a personal mindfulness course in the previous 12 months; or had previously trained to deliver MT to others.

### Procedure

Both schools and teachers were screened for eligibility, and the head teacher and participating teachers within the school provided informed consent. Once teachers had consented they were sent a link to an online questionnaire containing the baseline measures for the study (T0). Once at least 3 participating teachers within each school had completed this baseline assessment, and the head teacher had provided consent, the school was eligible to be randomized to 1 of 4 training routes. Schools (clusters) were randomized stratified by the number of teachers recruited in the school with the 2 strata defined by whether or not the school recruited more than 5 teachers. Randomization was conducted by Peninsula Clinical Trials Unit, which was not otherwise involved in the study.

#### Phase 1

Personal mindfulness courses commenced from early February 2016. Training start dates were staggered across schools to ensure training fitted with individual school calendars and participant commitments as far as possible. Start dates for self-taught courses were matched to instructor-led courses. Following completion of personal mindfulness courses, teachers were sent an email link to complete the post personal-training phase assessment (T1) which included measures assessing engagement with the MT course, their developing mindfulness skills, and their mental health and well-being. Completion of the T1 assessment marked the end of study phase 1, although teachers were encouraged to continue their personal mindfulness practice. Teacher mental health and well-being outcomes are reported in a parallel paper (Montero-Marin et al., under review).

#### Phase 2

Both 4-day and 1-day MT program training took place in July 2016 across 3 venues in England, with courses attended only by study participants. Four-day training involved release from school teaching commitments from a Wednesday through Friday, with training extending to the Saturday of the same week. One-day training involved release from school teaching commitments for a single Monday. Participant teachers always attended the same training course as other staff from their school. Members of the research team were present at each of the training venues to help support the teachers and give them more information about the study, especially around subsequent delivery of MT, through formal talks during the training and informally at social time. Following completion of program training, participants were sent a link to the T2 assessment. This included questions assessing engagement with the training course: teachers’ felt sense of preparedness, confidence and positivity about delivering the program to their pupils, their developing mindfulness skills, and their mental health and well-being (as reported elsewhere earlier).

Following training participants were given access to a closed online portal containing MISP program resources and were requested to deliver the program in the 2016 to 2017 academic year, and to video record their teaching. Cameras and instructions were provided by the research team. The project school liaison lead, LL, supported participants in timetabling of the program where participants requested this. Once teaching was completed, participants returned their video card, marking the end of their involvement in the study. Videos were processed to anonymize any pupils captured on film, prior to rating of teacher competency.

Both personal MT and program training were organized by the research team and delivered free of charge to participants. Participants were reimbursed for travel expenses to attend program training as well as their accommodation and subsistence expenses during training. Schools were also able to reclaim any supply costs incurred while their staff members were away from the school premises attending program training. Participants were compensated £100 in shopping vouchers for completion of all the questionnaires (this was split equally across the questionnaires and paid after each questionnaire was completed). Schools in which at least 1 teacher completed the study protocol were given £250 to spend on school resources at the end of the study.

### Model of Teacher Training

#### Phase 1–personal MT

The 2 forms of personal MT are described later, with full details provided in the Online Supplemental Material.

##### Instructor-led course

The instructor-led mindfulness course was based on the book *Mindfulness: Finding Peace in a Frantic World.*^26^ The course was delivered by trained and experienced mindfulness instructors over eight 90-minute group sessions, occurring approximately once per week. Participants read the course book^26^ alongside their group sessions. Attendance data for each course were collected.

##### Self-taught course

Participants allocated to self-taught training were provided with a course book, *Mindfulness: Finding Peace in a Frantic World.*^26^ Each participant was contacted prior to commencing the course and the importance of reading the whole course book and doing the associated activities was emphasized. Participants were asked to read the introductory chapters of the book and to commence the 8-week program outlined in the course book on a set date (usually the week following mail out of the books, and as far as possible contemporaneous with instructor-led groups).

#### Phase 2—program training

Standard 4-day training or condensed 1-day training was provided depending on the training route to which the school was randomized. Each comprised lesson modeling (in vivo and through viewing videos of experienced teachers), supported experience leading mindfulness practice and enquiry, and periods of personal mindfulness practice. Those on the 4-day training spent time in groups with the others from their school and participated in sessions to consider implementation of MT in their school. Teachers were not explicitly trained in how to work with adverse reactions.

### Sociodemographic Measures

#### Teacher and school characteristics

Data on teachers’ age, gender, and number of years in teaching were gathered alongside information on school type, school size (number of pupils), school quality (most recent government inspection outcome for state-funded schools, where available), and the percentage of pupils receiving free school meals (an indicator of school-level deprivation) for each participating school.

### Acceptability

#### Engagement with training

We monitored participants’ ongoing involvement with the study and recorded reasons for drop-out from the training protocol, and/or the study as a whole, wherever these could be obtained.

#### Adherence to personal MT

Participants in both instructor-led and self-taught MT reported: how much of the course book they had read; average days per week completing formal meditation practices, and average days per week completing informal mindfulness practices. In addition, participants in the instructor-led groups had session attendance recorded by their class instructor.

### Training Outcomes

#### Development of mindfulness skills

To establish whether core mindfulness skills were acquired through the training, we used the Five Facet Mindfulness Questionnaire–15 item Short Form. (FFMQ-SF).^27^ The short-form version of the FFMQ contains 3 items reflecting each of the 5 mindfulness facets (*observing, describing, nonreactivity, nonjudgment, and awareness*), which together form a total score. The internal reliability of the scale in the current sample was adequate (T0: alpha = 0.82, T1: alpha = 0.86, T2: alpha = 0.82).

#### Teaching competency

Teaching competency was assessed by independent raters, blind to training route, using a modified version of the Mindfulness-based Interventions Teaching Assessment Criteria (MBI-TAC).^28^ Two videoed lessons were anonymized for each participant teacher, to enable raters to view participants’ teaching skills in both the first and second halves of the program. Competency was initially rated on a scale from 1 (incompetent) to 6 (advanced). For statistical analyses, this scale was dichotomized into the categories *incompetent/beginner* (score of 1 or 2) versus *advanced beginner or above* (score of 3–6), with the latter category reflecting people judged to have a level of competency that is regarded as an adequate minimum to commence teaching. For clarity, and to prevent confusion with achievement of a “competent” rating on the MBI-TAC, we refer to this group as those who have reached the “minimum competency threshold,” and/or who achieve a competency rating of “advanced beginner plus.” For n* *=* *8 teachers (4% of those randomized, 8% of those rated for competency) teaching competency was assessed via review of a single videoed lesson due to technical errors in teachers’ recording of their sessions. Full details of the competency rating process including the training of raters is provided in the Online Supplemental Material.

### Statistical Methods

Baseline characteristics of the schools and teachers, and teachers’ self-rated preparedness, confidence, and positivity were summarized using means and standard deviations (or medians and interquartile ranges) for continuous variables and numbers and percentages for categorical variables. Missing data were not imputed. Baseline characteristics were summarized for those participants who were followed up and not followed up at each time point. At the end of phase 1, (T1) teacher mindfulness was compared between those allocated to instructor-led and self-taught MT training, using the intention-to-treat principle with teachers analyzed according to the trial arms their school was allocated to. Ongoing development of teacher mindfulness at T2 and teaching competency following program training were compared across all 4 arms, with instructor-led 4-day training compared with each of the lower intensity training arms: instructor-led 1-day training, self-taught 4-day training and self-taught 1-day training compared with the most intense, instructor-led 4-day route.

The FFMQ, as a continuous outcome, was compared between arms using random effects (“multilevel”) linear regression models to allow for correlation between responses from teachers within the same school (cluster). We ran unadjusted analyses and analyses adjusted for baseline FFMQ, teacher’s gender and age, and number of participating teachers in the school. The adjusted analyses are primary. We report the intracluster (intraschool) correlation coefficient for the unadjusted analyses.

Teaching competency was compared between training routes using random effects logistic regression models to allow for correlation between teachers within the same school (cluster). We ran unadjusted and adjusted analyses accounting for school quality rating (“requires improvement” or “inadequate,” compared with “outstanding,” “good,” or “not rated”). Since attrition from training was an important feasibility outcome and has important implications for the potential scalability and cost-effectiveness of a training pathway, 2 analyses were conducted. The first analysis included all participating teachers, irrespective of whether or not they completed the study protocol, with the assumption that those that did not complete the study protocol (ie, attend training and return a codeable video of their teaching) did not reach competency. The second analysis included only those teachers who completed the study protocol (ie, attended training and provided a codeable video of their teaching that could be used to rate competency). Analyses were performed using SPSS version 22 and R (version 3.5.1).

To assess cost-effectiveness, total training cost was calculated for each participant and included the cost of trainer time organizing, preparing and delivering the training, costs of course materials and training facilities, and supply cover costs associated with teachers’ attendance at training. We used actual costs incurred, so that if a teacher did not attend training, there were no associated costs. Unit costs, summarized in Table S3 in the Online Supplemental Material, were taken from trial management data and reported in U.K. pounds sterling for the financial year 2015 to 2016. Training costs, summarized as means and standard deviations, were combined with the analysis of teacher competency to determine the cost per teacher reaching the minimum competency threshold via each training route.

The primary economic analysis explored cost-effectiveness of the most intense, instructor-led, 4-day training route compared with each of the lower intensity training routes for all participating teachers. Cost-effectiveness was explored in terms of the proportion of teachers achieving competency and assessed through the calculation of incremental cost-effectiveness ratios , a summary statistic that presents the additional cost of 1 training route compared with another divided by additional effects generated.^29^ As cost data are commonly skewed, 1000 resamples were drawn from the data to generate a new distribution of mean costs and outcomes using the bootstrapping approach recommended for economic evaluations in the United Kingdom.^30^ Bootstrapped data were used to generate cost-effectiveness planes and cost-effectiveness acceptability curves that show the probability that the intensive training route is the optimal choice compared with each less intensive route, depending on a range of possible maximum values that a decision-maker might be willing to pay for an improvement in effects (the proportion of teachers reaching the minimum competency threshold).^31^ A secondary, scenario analysis explored cost-effectiveness for those teachers who completed the training protocol and submitted codeable teaching videos. Both analyses were adjusted for teacher gender and age, and number of participating teachers in the school.

## Results

### School and Participant Demographic Characteristics

In total, 43 schools were randomized and 206 teachers were recruited. Baseline school and teacher characteristics of participating teachers are shown in Table 1. In total, 13 schools (70 teachers) were randomized to instructor-led, 4-day training; 7 schools (35 teachers) to instructor-led, 1-day training; 10 schools (42 teachers) to self-taught, 4-day training; and 13 schools (59 teachers) to self-taught, 1-day training.

**Table 1.** Baseline School (Cluster) and Teacher Characteristics by Training Route.

	Self-taughtN = 23 SchoolsN = 101 Teachers	Instructor-led N = 20 SchoolsN = 105 Teachers	Instructor-led, 4-dayN = 13 SchoolsN = 70 Teachers	Instructor-led, 1-dayN = 7 SchoolsN = 35 Teachers	Self-taught, 4-dayN = 10 SchoolsN = 42 Teachers	Self-taught, 1-dayN = 13 SchoolsN = 59 Teachers
School characteristics						
Percent free school meals, median (IQR)	15.4 (11.9, 32.2)	22.9 (16.4, 39.1)	22.9 (16.4, 45.8)	25.1 (16.0, 34.0)	16.0 (12.1, 36.7)	14.8 (12.0, 28.1)
State schools, n (%)	21 (91)	17 (85)	13 (100)	4 (57)	9 (90)	12 (92.3)
Large schools, n (%)	13 (57)	10 (50)	8 (62)	2 (29)	5 (50)	8 (61.5)
Quality-rated as Good/Outstanding (state-funded schools only), n (%)	16 (76)	12 (71)	9 (69)	3 (75)	5 (56)	11 (91.7)
No. of participant teachers, median (IQR)	4 (3, 5)	5 (4, 6.3)	4 (4, 8)	5 (4.5, 5.5)	4 (3.3, 4.8)	5 (3, 5)
More than 5 teachers recruited, n (%)	11 (48)	11 (55)	6 (46)	5 (71)	3 (30)	8 (62)
Teacher characteristics						
Age, mean (SD)	40.1 (8.6)	38.0 (9.3)	36.5 (8.6)	40.9 (9.9)	40.5 (8.6)	39.8 (8.6)
Female, n (%)	80 (79)	80 (76)	55 (79)	25 (71)	37 (88)	43 (73)
Number of years teaching, median (IQR)	13 (8, 19)	10 (5, 17)	9 (5, 16)	10 (4, 18)	13 (8, 19)	12 (8, 20)
FFMQ–SF, mean (SD)	51.5 (7.3)	51.1 (6.8)	50.9 (6.5)	51.5 (7.3)	50.8 (7.8)	52.0 (6.9)

Abbreviations: FFMQ–SF, Five Facet Mindfulness Questionnaire–15 item Short Form; IQR, interquartile range.

Three schools in each of the instructor-led and self-help groups have missing data on the percentage of pupils claiming free school meals. Government quality ratings apply to state-funded schools only. Data are complete, in both groups, for all other baseline variables included.

### Study Flow

**Figure 1.**
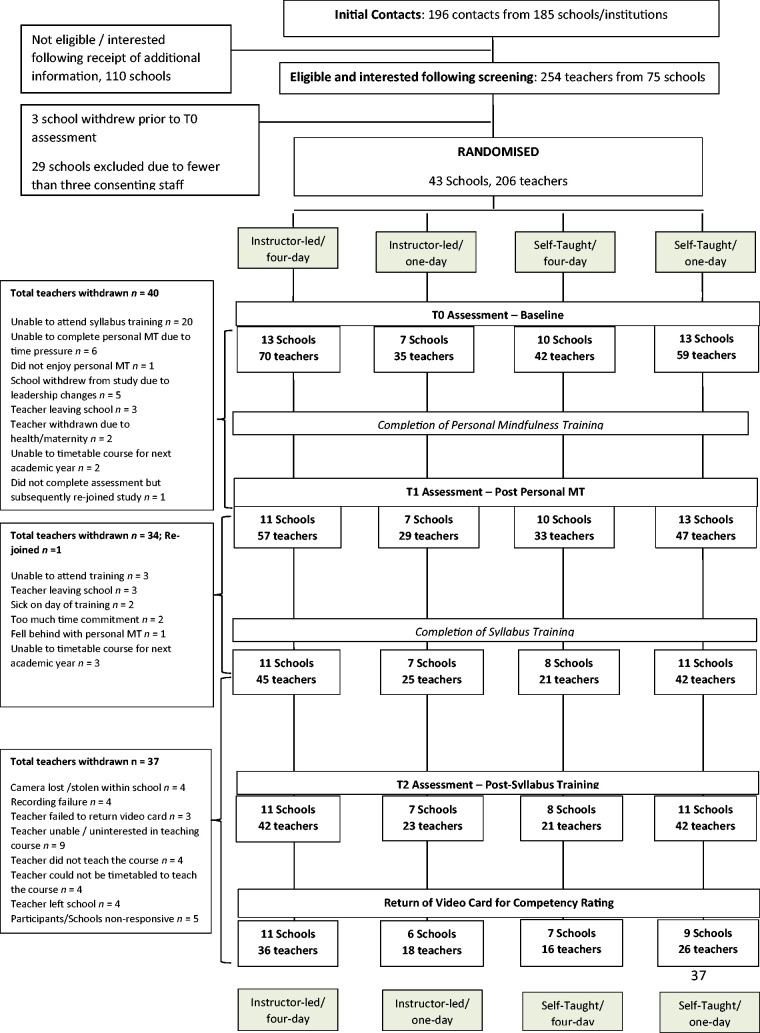
Participant Flow.

Study flow is shown in Figure 1. A comparison of baseline characteristics of teachers providing and not providing data at each assessment are provided in the Online Supplemental Material (Appendix 5 and Table S1), alongside reasons for attrition according to allocated training route (Appendix 3).

### Effectiveness: Development of Mindfulness Skills

Immediately following personal MT (T1) participants in the instructor-led routes had significantly higher scores on the FFMQ-SF, *M *=* *52.4, *SD *=* *7.1, than those in the self-taught routes, *M *=* *55.3, *SD *=* *6.8, *P *=* *.004, Glass’s Δ = −0.45, 95% confidence interval (CI) −0.75 to −0.16. Following program training (T2), there was some evidence of an overall effect of training route on FFMQ-SF (*P* = .05), which was most pronounced for the comparison between participants in the instructor-led, 4-day route and self-taught, 1-day route, Glass’s Δ = −0.59, 95% CI −1.04 to −0.13 (see Supplemental Material Table S4). These findings suggest that instructor-led training was more effective than self-taught training in promoting teachers’ personal mindfulness skills.

### Effectiveness: Teaching Competency

Considering all participants who were randomized to the study, only 29% of those randomized to instructor-led, 4-day training both completed the study protocol, returning a teaching video and were judged to have reached the minimum competency threshold. This figure fell to 24% of those randomized to self-taught, 4-day training, and 19% in those allocated to either of the 1-day training routes. Table 2 (part A) compares the competency of teachers in the 3 lower intensity training routes to those in the instructor-led, 4-day route, based on analyzing all participating teachers and assuming that those who did not complete the study protocol did not reach competency. There was little evidence of an overall effect on training route on the likelihood of reaching the minimum competency threshold, *P *=* *.07. Inspection of the data showed that participants in all 3 lower intensity training routes had a lower likelihood of reaching this threshold, than those in the highest intensity route: self-taught-4-day training, adjusted odds ratio (OR) = 0.67, 95% CI: 0.16 to2.74; instructor-led, 1-day training, adjusted OR = 0.51, 95% CI: 0.11 to2.42, and self-taught, 1-day training, adjusted OR = 0.47, 95% CI: 0.12 to1.82.

**Table 2.** Comparison of Routes in Terms of Proportion of Teachers Reaching the Minimum Competency Threshold (Advanced Beginner or Above) Among Those Who Were Randomized to One of the Four Training Routes (A), and Those Who Completed the Training Protocol Submitting a Teaching Video (B).

Training Group	n (%) (Advanced Beginner or Above)	Unadjusted OR^a^			Adjusted OR^b^		
		Estimate	95% CI	*P* Value	Estimate	95% CI	*P* Value
A. Randomized participants							
Instructor led 4 day (N = 70)	20 (29)	Ref		.8	Ref		.7
Self-taught 4 day (N = 42)	10 (24)	0.74	0.18–2.98		0.67	0.16–2.74	
Instructor led one-day (N = 35)	6 (17)	0.56	0.12– 2.63		0.51	0.11–2.42	
Self-taught one-day (N = 59)	10 (17)	0.55	0.15–2.05		0.47	0.12– 1.82	
B. Those submitting videos							
Instructor led 4 day (N = 36)	20 (56)	Ref		.4		Ref	.4
Self-taught 4 day (N = 16)	10 (63)	1.42	0.29–6.90		1.31	0.28–6.19	
Instructor led 1-day (N = 18)	6 (33)	0.42	0.09–2.03		0.43	0.09– 1.98	
Self-taught 1-day (N = 26)	10 (38)	0.50	0.13–1.95		0.43	0.11–1.73	

Abbreviations: CI, confidence interval; OR, odds ratio.

In Section A, those teachers without codeable teaching videos are assumed to have not reached the minimum competency threshold.

a From a model including training route variable only.

b From a model including training route variable and Quality rating (requires improvement/inadequate, compared with outstanding, good, not rated).

Considering only those participants who completed the study protocol and returned a teaching video, 56% of those who followed the instructor-led, 4-day training route were judged to have reached the minimum competency threshold, compared with 63% of those who followed the self-taught, 4-day training route, 33% of those who followed the instructor-led, 1-day training route and 38% of those who followed the self-taught, 1-day route. Table 2 (part B) compares the competency of teachers in the 3 lower intensity training routes to those in the instructor-led, 4-day route, based on analyzing those participants who returned a teaching video. There was little evidence of an overall effect of training route on the likelihood of reaching the minimum competency threshold, *P *=* *.40. Inspection of the data suggested that participants in the both 1-day routes had a lower likelihood of reaching this threshold: adjusted OR = 0.43, 95% CI: 0.09 to1.98 and adjusted OR = 0.43, 95% CI: 0.11 to1.73 for the instructor-led and self-taught 1-day routes, respectively, than participants in the instructor-led, 4-day route. There was also a 7% difference between the proportion of teachers achieving the minimum competency threshold in the instructor-led, 4-day training route and the self-taught, 4-day training route, favoring the latter. However, the wide confidence intervals around the odds ratio (OR (1.43; 95% CI = 0.29–6.90) suggest that this difference should be interpreted with caution.

### Acceptability

There was gradual but significant participant attrition in all 4 training routes over time (See Figure 1). In each, the main reason for attrition was an inability of participants to attend MT program training, with few participants reporting a lack of acceptability of personal mindfulness courses as a reason for attrition. The majority of reasons given were of a practical and logistical nature.

From T0 to T1 (pre- to postpersonal mindfulness courses) participant retention was equivalent across the 4 study arms (81% instructor-led, 4-day; 82% instructor-led, 1-day; 78% self-taught, 4-day; and 80% self-taught 1-day). There was little difference between the 2 forms of personal MT in reported levels of engagement with course content. In total, 77% of participants allocated to instructor-led training and 81% of those allocated to self-taught training, who responded to the T1 assessment, reported reading at least half the course content provided by the *Finding Peace in a Frantic World* book. Eighty-nine percent of participants in both instructor-led and self-taught training reported completing assigned home practice on 3 or more days per week. Informal home practice was reported to have been completed by 92% of instructor-led and 85% of self-taught participants on 3 or more days per week.

Class attendance for those allocated to instructor-led training was also recorded, with data referring to full attendance at sessions. Of those who provided T1 data, 73% attended at least 7 to 8 sessions, 22% attended between 4 and 6 sessions, and 5% attended < 4 sessions. Nineteen participants in the instructor-led route dropped out before the T1 assessment. Of these, 4 attended at least 7 to 8 sessions, 3 attended 4 to 6 sessions, and 12 attended <4 sessions. Many of these latter participants withdrew from personal MT when it became clear they would not be able to continue with further aspects of the study protocol.

There was some indication of differential attrition from the T1 assessment point onwards. Level of attendance at 1-day training (71% of those allocated) was higher than 4-day training (59% of those allocated). Retention of teachers to program training was particularly poor (50% of those allocated) in those who were allocated to the self-taught, 4-day route. Loss of whole schools (no teachers within a school providing video data due to whole school withdrawal, or cumulative individual attrition) was greater in the self-taught than instructor-led routes. The self-taught, 1-day route lost 4 schools and the self-taught, 4-day route lost 3. In comparison, the instructor-led, 1-day route lost 1 school and the instructor-led, 4-day route lost 2.

Finally, there was also some indication that the different training routes resulted in different proportions of individual teachers completing the full study protocol. In particular, inspection of Figure 1 suggests that participants in the instructor-led, 4-day and instructor-led, 1-day routes were more likely to complete the study protocol, and return videos of their delivery of the program within their school (51% in both groups), than participants in the self-taught, 4-day (38%) and self-taught, 1-day (44%) routes.

### Costs and Cost-effectiveness

Table 3 reports unadjusted mean training costs per teacher, competency rates, and cost per teacher reaching the minimum competency threshold for each training route. Among those who were randomized (primary economic analysis) mean training costs were lower for 1-day routes: self-taught, 1-day, *M* = £254.07, *SD* = £169.55, and instructor-led, 1-day, *M* = £530.22, *SD* = £182.93, than for 4-day routes: self-taught, 4-day *M* = £580.76, *SD* = £599.96, and instructor-led, 4-day, *M* = £987.28, *SD* = £556.18.

**Table 3.** Comparison of Unadjusted Mean Costs, Competence Rates, and Costs Per Teacher Reaching the Minimum Competency Threshold (Advanced Beginner or Above) Among Those Who Were Randomized (Primary Analysis) and Those Who Completed Study Protocol (Scenario Analysis).

Training Group	Primary Analysis *(Among Those Who Were Randomized)*				Scenario Analysis *(Among Those Who Completed Study Protocol)*			
	N	Cost Mean (SD)	Proportion reaching minimum competency threshold	Cost per teacher minimum competency threshold	N	Cost Mean (SD)	Proportion reaching minimum competency threshold	Cost per teacher reaching minimum competency threshold
Instructor-led, 4-day	70	£987.28 (£556.18)	29%	£3455.46	36	£1359.67 (£233.28)	56%	£2447.41
Self-taught, 4-day	42	£580.76 (£599.96)	24%	£2439.20	16	£1151.52 (£231.92)	63%	£1842.44
Instructor-led, 1-day	35	£530.22 (£182.93)	17%	£3092.94	18	£636.13 (£77.79)	33%	£1908.38
Self-taught, 1-day	59	£254.07 (£169.55)	17%	£1499.03	26	£352.86 (£77.25)	38%	£917.44

Unadjusted mean costs per teacher reaching the minimum competency threshold increased with increasing intensity of training ranging from £1499.03 in the least intense training route (self-taught, 1-day) to £3455.56 in the most intense route (instructor-led, 4-day). The higher cost per teacher reaching this threshold, compared with the cost per teacher noted earlier, reflect the costs associated with training teachers who did not achieve this threshold and teachers who completed parts of the training protocol, and thus incurred training costs, but subsequently dropped out.

**Figure 2.**
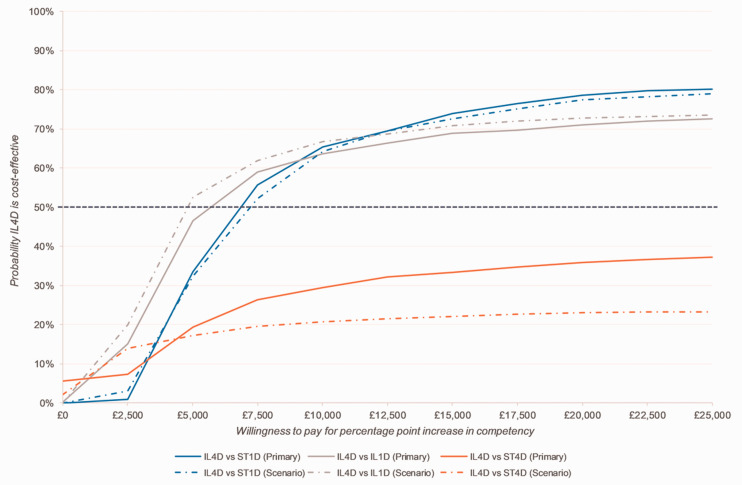
Cost-effectiveness Acceptability Curve Showing the Probability That Standard Instructor-Led, 4-Day Training is Cost-effective Compared to Less Intensive Training Routes for Different Values of Willingness to Pay for Percentage Point Increase in Teachers Reaching the Minimum Competency Threshold (Advanced Beginner or Above) Among Those Who Were Randomized (Primary Analysis) and Those Who Completed the Study Protocol (Scenario Analysis). IL4D, instructor-led, 4-day; ST4D, self-taught, 4-day; IL1D, instructor-led, 1-day; ST1D, self-taught, 1-day.

After adjusting for teacher’s gender and age, and number of participating teachers in the school, results of the primary cost-effectiveness analysis, considering all teachers who were randomized, suggest that instructor-led, 4-day training is not only more costly but also more effective than any of the less intensive routes (Table 3). The cost-effectiveness acceptability curve (Figure 2) indicates that instructor-led, 4-day training has a lower probability (<50%) of being cost-effective than self-taught, 4-day training, irrespective of the level of willingness to pay for improvements in the proportion of teachers reaching the minimum competency threshold teachers (orange line) and only has a greater probability of being cost-effective than 1-day routes (blue and grey lines) at willingness to pay thresholds above £5,000 for a percentage point increase in competency. Cost-effectiveness analyses considering only those teachers completing the study protocol (scenario analysis; dashed lines in Figure 2) showed similar results to the primary analysis. Full details of the cost-effectiveness results, including the scenario analysis, are provided in Online Supplemental Material Appendix 4.

## Discussion

This study compared a current established method for training school classroom teachers to teach a mindfulness program (.b, developed by the “Mindfulness in Schools Project), with 3 more scalable alternatives. These alternatives were constructed through the combination of instructor-led or self-taught personal MT, and 4-day or 1-day program training. Our main outcome of interest was teaching competency on not only the completion of training (quantified as the proportion of teachers reaching a minimum competency threshold of advanced beginner on the MBI-TAC teach) but also the extent to which the various training routes increased teachers’ personal mindfulness skills, and the training routes’ relative acceptability and costs.

To the best of our knowledge, no previous research has examined the impact of different training routes on teaching competency in any MT program. The descriptive statistics and odds ratios suggest that when comparing outcomes for all those who started the 4 training pathways, competency levels were greater among those who received the existing training, when compared with those who received all three lower intensity alternatives. However, these differences were not statistically significant and a larger adequately powered trial would be required to extend and replicate these findings. Notably, our findings suggest that even the standard training route leaves a substantial proportion of teachers requiring further support or experience to improve their MT teaching to a sufficient level of competency.

Turning to acceptability, we observed high levels of attrition from the study protocol in all 4 training routes, although there was also some indication that this attrition differed according to training route in meaningful ways. Some of the attrition we observed may reflect “natural selection” of teachers, with those who did not feel competent to teach, or who were no longer interested in teaching MT after experiencing it for themselves. Such self-selection is likely to be an important part of any training process. Data also suggested that attrition related substantially to the demands of training on staff members and schools, difficulty scheduling the program within the school timetable, and movement of teachers within job roles. Despite these pressures, however, there was no clear indication that reducing training demands by replacing an instructor-led course with a self-taught course would enhance completion of the training protocol. Likewise, although attendance at MT program training was cited as a reason for discontinuation and was more pronounced among those allocated to 4-day training, it was also a common reason for attrition among those required to miss only 1 school day.

Inspection of the attrition data suggested that those who had received an instructor-led personal mindfulness course were more likely to go on to complete the entire study protocol, and thus to deliver the MT program within their classrooms. The reasons for this are unclear. One possibility is that those who received an instructor-led course may have perceived MT to be of greater value, as a consequence of their greater personal benefit indexed by greater development of mindfulness skills, and thus have been more individually motivated to deliver it to their pupils. In addition, it is possible that instructor-led mindfulness courses may have increased teachers’ confidence, through greater exposure to modeling of mindfulness teaching. Finally, the presence of an experienced mindfulness instructor delivering training at each school over an 8-week period may have increased the wider schools’ exposure and commitment to MT, and thus reduced school-level barriers to subsequent delivery of MT to pupils. Further exploration of the role of exposure to personal MT within a school community on implementation trajectories of MT within schools is something that is being exploring through process evaluation in an ongoing randomized controlled trial (Kuyken et al., 2017).

Our economic analysis indicated that, as expected, the mean costs for 1-day program training routes were lower than for 4-day training routes, and that when considering the mean costs of training as a function of the number of the proportion of teachers reaching the minimum competency threshold costs were higher for instructor-led than self-taught training routes. Cost-effectiveness analyses suggested a high probability of self-taught, 4-day training being cost-effective compared with instructor-led, 4-day training but was less clear for comparisons between other routes. One-day program training routes would save considerable resources, but to the detriment of competency. Self-taught personal MT combined with 4-day program training may be cost-effective relative to instructor-led personal MT. However, these analyses focus only on teaching competency and do not consider personal benefits for teachers, in terms of acquisition of mindfulness skills, which were superior in the instructor-led routes. In addition, early attrition was highest in the self-taught, 4-day route, which might pose challenges in schools in which there was only a limited pool of potential MT teachers. While wide confidence intervals around estimates preclude firm conclusions, the data suggest that attempting to increase scalability of MT teacher training by reducing training intensity requires careful consideration. Indeed, the standard program studied is already diluted over what is considered necessary to train mindfulness teachers in other contexts,^13^ and the outcomes suggest greater investment in training may be required to reach an optimal outcome of getting all teachers ready to teach the program competently from their first delivery.

### Study Limitations

As a first study in this area we conceptualized it as exploratory and as such it was underpowered to fully investigate all our questions. Thus our findings should be considered preliminary, hypothesis generating and the basis for future, much needed research. In addition, there are a number of specific considerations relating to the nature of the program studied; the ways the research context differs from natural implementation, and the measure of competency used, which all need to be taken into account in interpreting the results.

First, this study focused on training teachers to deliver one particular MT program. While the .b program has started to be the focus of research and implementation internationally^32–35^ its specific content and pedagogy (including scripted lessons, supporting animations, substantial psychoeducational content, and relatively brief periods of mindfulness practice) may influence the effectiveness of condensed training in both positive and negative ways. Generally speaking, lower intensity and more standardized interventions are more scalable. For schools-based universal MT, it is not clear what the optimal balance is in intensity and standardization of program and therefore to what extent these findings would relate to preparation of teachers for other MT programs or indeed trainings that bypass teachers altogether and provide young people directly with MT, via digital programs for example.

Second, the experiences of teachers and schools participating in this research study differed from natural implementation in several ways. For example, initial motivation to participate in the study often came from individual teachers within a school, who despite their interest, were naïve to mindfulness. Thus, there may have been relatively little institutional buy-in to the study or internal support for teachers wishing to deliver the program, which is likely to have contributed to difficulties teachers faced in delivering the program following training. In contrast, research suggests that natural implementation often relies on grassroots champions within schools who have a strong commitment to MT, engage wider staff and train teachers.^7–9^. In addition, although the costs and organizational demands of training were borne by the study rather than teachers or school, reducing resourcing barriers, teachers consequently had less flexibility with regards to when they accessed program training, and difficulties with attendance at this training were a significant source of attrition. The study protocol also deviated in one respect from the recommended training route of MISP. Within the MISP pathway, it is recommended that teachers leave a period of 6 months from commencement of a personal mindfulness course to completion of syllabus training. Participants in this study had a slightly shorter interval (4–5 months) between the 2 phases of MT. The impact of this is unknown, since the value of a greater interval between a personal mindfulness course and subsequent MT program training is likely to depend on the quality of independent mindfulness practice in the intervening period. In addition, longer intervals between training phases are also likely to increase attrition. Finally, the fact that teachers were required to seek opt-out consent from pupils’ parents to video record their teaching, might have placed an additional barrier in the way of delivery of the program.

Third, we assessed teachers’ competency on their first ever delivery of the MT program. It is unknown whether, as a group, teachers’ competency would increase over time with repeated delivery of the program, or whether it would in fact deteriorate as they began to forget their learning from their personal mindfulness course and MT program training. It has been observed in previous research analyzing the validity of the MBI:TAC that competence of students in later years of a Master’s program improves.^36^ Our study would have been strengthened by a further assessment of competency at a subsequent delivery of the program, and a consideration of how competency trajectories relate to prior training and ongoing professional development. We report data on overall competency and dichotomized MBI-TAC ratings at the beginner/advanced beginner level in order to identify teachers who had reached what is widely regarded as a threshold of minimum competency to commence teaching under supervision. However, it is plausible that training routes may have different impacts on different domains of competency, and that teachers might improve in some domains with further independent practice, for example, becoming more familiar with course materials and lesson structures, but might diminish in competency on others, with aspects such as embodiment of mindfulness potentially relying on ongoing personal practice and diminishing over time in its absence. Finally, although we put in place robust procedures to rate the competency of teachers, using a measure based on consensus concerning what good MT teaching looks like, generally and within schools specifically, it remains unknown how the measure of teacher competency we used relates to pupils’ benefit from MT teaching. Although it might be assumed that teachers who are rated as more competent would produce better outcomes for pupils, to date research into the relationship between teaching competency and participant outcomes in MBIs is at an early stage. There is only limited evidence of associations between instructor competency and participant benefit in other mindfulness-based programs^37^ and this is an important question to be explored in future research. The same argument extends to associations between instructor competency and potential harms for the children they go on to teach. We know little about typical effects of brief mindfulness practices in young people, nor the ability of teachers to anticipate or mitigate any difficulties children have with the this MT curriculum. The potential for harm is both an ethical imperative and an emerging area of research. With all such universal social–emotional skills generally and mindfulness curricula specifically, it is important to consider how they are implemented so they are embedded alongside school safeguarding procedures and mental health services in ways that maximize their reach, effectiveness, cost-effectiveness, and minimize harm.

### Implications and Future Directions

This first study of different models of teacher training in a mindfulness program generates some important avenues for future research. First, the standard “best practice” training is producing only modest levels of competency. Teacher training in the program might benefit from (1) more intensive personal MT; (2) greater rehearsal, with formative feedback to teachers during the program training; (3) mentoring and supervision of teachers as they start to offer MT to children in schools; and (4) greater support to schools in implementation generally. All these have implications for resourcing, in terms of training, teacher time, costs, and school timetables, but we hypothesize they may be necessary to ensure good quality delivery of MT in the classroom. Implementing these training enhancements in ongoing research^25^ will provide evidence in due course on whether teaching competency is enhanced through these measures, and how this relates to outcomes for children, teachers, and schools.

The findings also have implications for the training of mindfulness teachers more broadly. In the United Kingdom, Mindfulness-based Cognitive Therapy (MBCT) has been recommended as an approach for adults at risk for depressive relapse to learn skills to stay well within the National Health Service since 2004 with the guidelines updated in 2009.^37^ In 2017, a U.K. national training program for mindfulness teachers was published based on extant best practice.^39^ Within this context, the first cohort of cognitive-behavioral therapists have been trained and using the same measure of competency (MBI-TAC), >90% have been assessed as competent using the same measure and benchmark (advanced beginner level or above) as in this study. This suggests that it is possible to reach a threshold of adequate minimum competency through scalable training for those wishing to deliver MT in other public sector contexts. However, while there has for some time been an emerging consensus about how best to train mindfulness teachers to deliver mindfulness curricula^13^ much of the thinking in this area has originated from the use of MT in health care. As MT enters spheres such as education, the workplace, or community health-care systems and is taught by people with a broader array of professional backgrounds, organizational contexts and expectations regarding training and development, very real tensions can emerge between making MT accessible and scalable, and retaining quality. Research of the kind reported here has the promise of turning what could be seen as trade-offs into creative dialectical tensions that may lead to effective innovation in training models in the future.

## Supplemental Material

sj-pdf-1-gam-10.1177_2164956120964738 - Supplemental material for Training School Teachers to Deliver a Mindfulness Program: Exploring Scalability, Acceptability, Effectiveness, and Cost-effectiveness Click here for additional data file. Supplemental material, sj-pdf-1-gam-10.1177_2164956120964738 for Training School Teachers to Deliver a Mindfulness Program: Exploring Scalability, Acceptability, Effectiveness, and Cost-effectiveness by Catherine Crane, PhD, Poushali Ganguli, PhD, Susan Ball, MSc, Laura Taylor, PhD, Sarah-Jayne Blakemore, PhD, Sarah Byford, PhD, Tim Dalgleish, PhD, Tamsin Ford, PhD, Mark Greenberg, PhD, Willem Kuyken, PhD, Liz Lord, MA, Jesus Montero-Marin, PhD, Anna Sonley, MEd, Obioha C Ukoumunne, PhD and J Mark G Williams, PhD in Global Advances in Health and Medicine
