# Supplementary material for: Training School Teachers to Deliver a Mindfulness Program: Exploring Scalability, Acceptability, Effectiveness, and Cost-effectiveness
Source: Glob Adv Health Med. 2020 Dec 15;9:2164956120964738. doi: 10.1177/2164956120964738 (PMC7745556; doi:10.1177/2164956120964738)
Supplement: sj-pdf-1-gam-10.1177_2164956120964738 - Supplemental material for Training School Teachers to Deliver a Mindfulness Program: Exploring Scalability, Acceptability, Effectiveness, and Cost-effectiveness [file sj-pdf-1-gam-10.1177_2164956120964738.pdf]

Training School Teachers to Deliver a Mindfulness Program: Exploring Scalability, Acceptability, Effectiveness and Cost-Effectiveness

Online Supplementary Materials

Online Appendix 1. Full Description of Elements of the Mindfulness Training Routes

Online Appendix 2. Assessment of Teaching Competency

Online Appendix 3. Reasons for Participant Withdrawal, by Study Phase and Study Arm

Online Appendix 4. Full Results of Cost-Effectiveness Analysis

Online Appendix 5. Supplementary Tables

Online Appendix 1

Full Description of Elements of the Mindfulness Training Routes

Phase 1 – Personal Mindfulness Training.

Instructor-led Course. The instructor-led manualized mindfulness course, developed by Chris Cullen, Mark Williams and Danny Penman, was based on the book *Mindfulness: Finding Peace in a Frantic World* (Williams & Penman, 2011). The course was delivered over eight 90-minute sessions, scheduled at the end of the school day (twilight session), occurring approximately once per week. Within each school, teachers participated as a single group. Group sizes ranged from three to nine participants. Participants used the book by Williams and Penman (2011) to guide them through the course, reading chapters corresponding to each of the eight instructor-led sessions. Participants also received a booklet with supporting materials and place to record their home practice.

Each course was delivered by one of a team of experienced mindfulness instructors who met good practice guidelines (http://mindfulnessteachersuk.org.uk/#guidelines). All instructors had additionally received a two-day training workshop in the program, led by the developer of the face-to-face version of the program, Chris Cullen and also input regarding expectations and practicalities of working within a school setting. They each received an initial supervision session with LL and then regular supervision and 'helpful tips when working in schools' emails during course delivery to address any issues that arose in groups (for example managing teachers at different levels of seniority within the group). Participants were given access to the audio files accompanying the course via CD, MP3 and a freely available App developed by the book publisher to accompany the course book.

Self-taught Course. Participants in the self-taught arm of the study were provided with a course book, *Mindfulness: Finding Peace in a Frantic World* (Williams & Penman,

2011), and given access to the audio files accompanying the course via CD, MP3 and the associated App. Each participant was contacted prior to commencing the course and the importance of reading the whole course book was emphasized. Participants were asked to read the introductory chapters of the book and to commence the 8-week program outlined in the course book on a set date (usually the week following mail out of the books, and as far as possible contemporaneous with instructor-led groups). Participants also received a booklet with supporting materials and place to record their home practice. Participants were advised to follow the program over an 8-week period and were free to contact the research team if they had any difficulties accessing audio files or questions about the study. However no personalised support concerning course completion was provided. Teachers were not instructed either for or against discussing course content with colleagues also enrolled in the study.

Phase 2 – Program Training.

Four-day Training. The four day training consisted of the following elements: *Lesson Modelling* - .b trainers 'taught' each of the 10 lessons fully, with participants encouraged to engage with the lessons as if they knew nothing about mindfulness (i.e. to take the role of a student and engage with the lesson in real-time). Each taught lesson was then followed by periods of discussion during which the lesson pedagogy was explored; *Leading Practice and Enquiry* – these sessions explored what it was like to lead mindfulness practice and enquiry with young people, some of the typical issues and challenges that could arise, and how teaching mindfulness to young people might differ from teaching mindfulness to adults; *Teach Back* – during these sessions attendees practised leading the mindfulness practices included in each lesson plan (nine teach-back sessions in total), as well as guiding the enquiry that followed. Trainers observed and provided supportive feedback on participants efforts; *Home Groups* – these sessions allowed attendees to work in smaller

groups and to discuss issues they may not have had the chance to raise in the larger group, as well as to discuss and plan how they envisaged mindfulness being implemented and supported in their school ('The School Challenge'); *Periods of Mindfulness Practice* – Extended periods of mindfulness practice were included throughout the training (six sessions in total) in order to allow participants to deepen their personal mindfulness practice over the four-days; '*Our Journey so Far'* – these smaller group work sessions involved creating posters charting the structure and flow of the .b lessons in order to understand the scaffolding and logic that lies beneath them; *Video clips* – observing what it is like to teach .b in the classroom – real lessons being taught to real classes.

One-day Training. The one-day training followed the four-day course with the following adaptations: *Lesson Modelling* – whilst two key lessons were taught in full, the remaining lessons were partially 'talked through' – with participants being given an overview and brief discussion of lesson content, rather than seeing each lesson being taught in full. *Leading Practice and Enquiry* – one opportunity was provided to discuss what it was like to lead mindfulness practice and enquiry with young people, some of the typical issues and challenges that could arise, and how teaching mindfulness to young people might differ from teaching mindfulness to adults; *Teach Back* –participants had a single opportunity to practice teaching a mindfulness practice and to receive supportive feedback; *Home Groups* – home group sessions were not included; *Periods of mindfulness practice* – two shorter periods of mindfulness practice were included in the training day; '*Our Journey so Far*' – this exercise was excluded from 1-day training; *Video clips* – participants watched a real lesson being taught to a real class and discussed their observations.

References

Williams, J.M.G., & Penman, D. (2011). *Mindfulness: A practical guide to finding peace in a frantic world*. Piatkus, UK.

Appendix 2

Assessment of Teaching Competency

Teaching competency was assessed using a modified version of the Mindfulness-based Interventions Teaching Assessment Criteria (MBI-TAC). The MBI-TAC was designed to support assessment of teacher adherence and competence when delivering Mindfulness-based Interventions (MBIs) (Crane et al., 2013; Crane et al., 2012). The general psychometric properties of the MBI:TAC have been previously tested (Crane & Kuyken, 2019). The Cronbach alpha values (mean .88, range across domains .84-92) are adequate with good inter-rater reliability (ICC = .81, $p < .01$), and adequate construct and concurrent validity when MBI-TAC rating is examined in relation to stage of training. Although used primarily to assess Mindfulness-based Stress Reduction (MBSR) and Mindfulness-based Cognitive Therapy (MBCT) teaching, it has also been used for other MBIs derived from these programs (Crane & Kuyken, 2019). The teaching addendum to the MBI-TAC (MBI-TAC TEACH, available from the authors on request) provided some adaptations to the MBI-TAC to make it suitable for assessment of MBIs delivered in school settings. The MBI-TAC TEACH was developed to be used alongside the MBI-TAC manual. But it adds additional information to rate school classroom teachers teaching mindfulness to young people in school contexts. It covers the same domains as the MBI-TAC, but within each domain identified issues that are specific to teaching mindfulness to children in school contexts. For example, in Domain 6 which concerns holding the group learning environment there is inclusion of teachers being aware of using school safeguarding procedures and classroom management. The MBI-TAC assesses teaching competency across six domains: Coverage, Pacing and Organisation of Session Program; Relational Skills; Embodiment of Mindfulness; Guiding Mindfulness Practices; Conveying Course Themes; Holding the Group Learning Environment, and an overall rating of teaching competency, which formed the primary outcome for the study.

Each domain and overall teaching competency is rated on the following scale: 1 – incompetent; 2 – beginner; 3 – advanced beginner; 4 – competent; 5 – proficient; 6 – advanced. For the purposes of the study reported, the overall six point rating was dichotimised at the incompetent or beginner / advanced beginner or above boundary. This is because people reaching advanced beginner level or above are generally regarded as having reached an adequate minimum competency threshold and able to begin teaching under supervision. In the context of a teacher delivering MT in schools this was considered to reflect a reasonable expected level of competency on first delivery of the program.

Independent raters who were experienced MBI teachers, experienced in the delivery of the .b MT program, and trained in use of the MBI-TAC received additional training in the administration of the MBI-TAC TEACH addendum in a two day workshop led by WK. Each domain of the MBI TAC TEACH was reviewed and joint ratings were conducted with the use of illustrative teaching video extracts, in order to establish initial consistency. Following the training workshop, all trainees independently rated videos of two participants delivering the .b course, viewing two sessions from each teacher, and rating the six competency domains as well as providing a composite rating based on an overall impression of teaching competency for each teacher. Videos for this phase were benchmarked through consensus rating by WK and LL (an experienced MBI-TAC trainer & rater, and an experienced teacher & .b instructor, respectively) with sessions selected to expose trainees to a broad range of classroom contexts and teacher competency levels. Raters participated in a video conference to review their independent ratings and explore discrepancies. Following this, the reliability of raters was assessed**.** Raters reviewed videos of a further three participant teachers and rated competency of each. Their ratings were compared to benchmark values (WK & LL) and an average disagreement score was calculated for each rater. All six raters had an average discrepancy of less than one point on the MBI-TAC Teach from the benchmarks, indicating a

high degree of consistency between independent raters and the benchmark. During subsequent independent video coding, all raters participated in periodic video conferences to review processes and raise any issues arising during coding.

Raters reviewed two anonymised lessons to assess participants' teaching skills in both the first and second halves of the course, and to view sessions which contained activities and elements addressing all the domains on the MBI-TAC TEACH. Sessions for rating were randomly assigned from Sessions 3&6, 4&6, or 3&7. In some instances, teachers did not capture all lessons on video, because they forgot, mislaid their camera, experienced technical difficulties or had changes in scheduling that meant they could not access the camera at the required time. Where randomly assigned sessions were not available, Session 8 was substituted if available. For *n* = 88 participants (43% of those randomised, 92% of those rated for competency) two teaching videos were assessed, but for *n* = 8 cases (4% of those randomised, 8% of those rated for competency) competency had to be assessed via review of a single videoed session.

References

1. Crane Crane, R.S., Soulsby, J. G., Kuyken, W., Williams, J. M. G., Eames, C., Bartley, T… & Silverton, S. (2012). *The universities of Bangor, Exeter & Oxford, Manual of the Mindfulness-based Interventions Teaching Assessment Criteria (MBI-TAC).* Accessed at http://mbitac.bangor.ac.uk/documents/MBITACmanualsummaryandaddendums0517.pdf, 3rd July 2019.
2. Crane, R. S., Eames, C., Kuyken, W., Hastings, R. P., Williams, J. M., Bartley, T., Evans, A., Silverton, S., Soulsby, J. G., & Surawy, C. (2013). Development and

validation of the mindfulness-based interventions teaching assessment criteria (MBI-TAC). *Assessment*, *20*, 681-688.

.

3. Crane, R., & Kuyken, W. (2019). The Mindfulness-Based Inverventions: Teaching Assessment Criteria (MBI:TAC): reflections on implementation and development. Current Opinion in Psychology, 28, 6-10.

Appendix 3

Reasons for Participant Withdrawal, by Study Phase and Training Route

In the tables that follow we provide reasons for attrition broken down by study phase and training route. It should be noted that this information is accurate to the best of our knowledge and is provided to give an overview of typical reasons for attrition in each study arm. However we relied on participants', and in some instances their colleagues', reports on reasons for attrition, and this should be taken into account in interpreting the data.

Reasons for Participant Withdrawal, T0 to T1

Instructor-Led, Four-day Training	N
Unable to attend program training	5
Unable to complete personal mindfulness training due to time commitments	1
Single whole school withdrew from study due to leadership changes	5
Moved school	1
Withdrawn due to health issues	1
Total	13

Instructor-Led, One-day Training	N
Unable to attend program training	4
Did not enjoy personal mindfulness training	1
Moved school	1
Total	6

Self-taught, Four-day Training	
Unable to attend program training	4
Did not have time to continue participation in personal mindfulness training	4
Unable to teach course in subsequent academic year	1
Total	9

Self-taught, One-day Training	N
Unable to attend program training	7
Did not have time to continue participation	1
Did not complete assessment, but rejoined program training	1
Teacher changed schools	1
Maternity leave	1
Unable to teach course in subsequent academic year	1
Total	12

Reasons for Participant Withdrawal, T1 to Program Training

Instructor-Led, Four-day Training	N
Unable to attend training	8
Unable to timetable course	1
Too much time commitment	1
Moving schools	2
Total	12

Instructor-Led, One-day Training	N
Unable to attend training	4
Total	4

Self-taught, Four-day Training	
Unable to attend training	8
Fallen behind with personal mindfulness and did not want to continue	1
Sick during program training	1
Moving schools	1
Insufficient time	1
Total	12

Self-taught, One-day Training	N
Unable to teach next year	2
Sick on training day	1
Unable to attend syllabus training	3
Total	6

Note: Six participants withdrew and one returned having missed the T1 data point

Reasons for Participant Withdrawal following Program Training

Instructor-Led, Four-day Training	N
Camera and memory card stolen within school	1
Memory card and camera lost within school	3
Teacher reported teaching course but did not return video card	1
Teacher could not be timetabled to teach course	4
Total	**9**

Instructor-Led, One-day Training	N
Did not teach course	3
Camera/recording failure	3
Teacher did not return memory card	1
Total	7

Self-taught, Four-day Training	N
Teacher did not teach course	1
Teacher left the school	1
Participants / School were non-responsive	3
Total	5

Self-taught, One-day Training	
Teacher on sabbatical / teacher left school	3
Camera/recording failure	1
Not able or interested in teaching course	9
Participants /School were non-responsive	2
Teacher reported teaching course but did not return a memory card	1
Total	16

Appendix 4

Full Results of Cost-Effectiveness Analysis

Results of the primary cost-effectiveness analysis, considering all teachers that were randomised, suggest a larger difference in both costs and outcomes between the standard training route, instructor-led, four-day, and one-day training routes compared to the self-taught, four-day training route (Table S3). The comparison between instructor-led, four-day and self-taught, one-day shows an adjusted difference between costs of £729.80 and adjusted difference between effects (proportion of competent teachers) of 12.15%, generating an ICER of £60.04 per percentage point increase in proportion of teachers deemed competent. Comparing instructor-led, four-day with instructor-led, one-day shows a difference in costs of £479.14 and a difference in effects of 9.81%, resulting in an ICER of £48.53 per percentage point increase in proportion of teachers reaching the threshold for adequate minimum competency. The comparison of instructor-led, four-day with self-taught, four-day shows relatively smaller differences in costs (adjusted mean difference: £361.21) and effects (adjusted mean difference: -3.62%), resulting in an ICER of -£99.73 per percentage point increase in proportion of competent teachers. However, in the scenario analysis the comparison of instructor-led, four-day with self-taught, four-day shows a difference in costs of £281.09 and a difference in effects of -17.75%, due to the higher rates of competency for self-taught, four-day route in this analysis (63% competent in scenario analysis versus 24% competent in the primary analysis; Table 7). Comparison of instructor-led, four-day with both one-day routes for the scenario analysis shows similar results as in the primary analysis.

The cost-effectiveness plane for the primary analysis (Figure S1) shows that most scatter points for comparisons between instructor-led, four-day training and self-taught, one-day training (blue), and between instructor-led, four-day training and instructor-led, one-day

training (grey line) fall mainly in the northeast quadrant, indicating that instructor-led, four-day training is more costly, but also more effective. Comparing instructor-led, four-day training with self-taught, four-day training however results in more scatter points falling across the northeast (instructor-led, four-day training more costly and more effective) and northwest (instructor-led, four-day training more costly and less effective) quadrants. This indicates a lower probability of instructor-led, four-day training being cost-effective compared to self-taught, four-day training, than of instructor-led, four-day training being cost-effective compared to either of the one-day routes.

The associated cost-effectiveness acceptability curves (Figure S2) suggest that the probability of instructor-led, four-day training being cost-effective compared to one-day training, whether with instructor-led or self-taught MT (blue and grey lines), is heavily dependent on society's willingness to pay for an increase in competent teachers; the higher the willingness to pay, the higher the probability of instructor-led, four-day training being cost-effective compared to the one-day training options. In contrast, the results comparing instructor-led, four-day training and self-taught, four-day training (orange line) suggests that the probability of instructor-led, four-day training being cost-effective is below 50% irrespective of willingness to pay. In other words, there is a higher probability of self-taught, four-day training being cost-effective compared to instructor-led, four-day training.

The scenario analysis, considering participants that completed the study protocol and submitted codeable videos shows greater variability in estimates of costs and effects for all three comparisons (Figures S3 and S4). However, results are very similar to those in the primary economic analysis, with the cost-effectiveness of instructor-led, four day training compared to either instructor-led or self-taught, one-day training, being uncertain and dependent on willingness to pay, whilst the comparison with self-taught, four-day training is

clearer and suggests a greater probability of self-taught, four-day training being cost-effective compared to instructor-led, four-day training.

Appendix 5

Supplementary Tables and Figures

Supplementary Table Legends

Table S1. Comparison of baseline characteristics of those providing and not providing data at each time point.

Table S2. Comparison of teacher primary outcomes and mechanism measures at post-intervention follow-up (T2), adjusting for baseline (T0).

Table S3. Summary of costs used in economic analyses.

Table S4. Differences in mean costs and effects (proportion of teachers reaching minimum competency threshold) among those who were randomised (primary analysis) and those who completed study protocol (scenario analysis).

Supplementary Figure Legends

Figure S1 Bootstrapped mean differences in costs and effects (proportion of teachers reaching the minimum competency threshold) among those who were randomised (primary analysis).

Figure S2. Cost-effectiveness acceptability curve showing the probability that standard training (IL4D) is cost-effective compared to less intensive training routes for different values of willingness to pay for percentage point increase in teachers reaching the minimum competency threshold among those who were randomised (primary analysis).

Figure S3. Bootstrapped mean differences in costs and effects (proportion of teachers reaching the minimum competency threshold) among those who completed study protocol (scenario analysis).

Figure S4. Cost-effectiveness acceptability curve showing the probability that standard training (IL4D) is cost-effective compared to less intensive training routes for different values

of willingness to pay for percentage point increase in teachers reaching the minimum competency threshold among those who completed study protocol (scenario analysis).

Table S1. Comparison of baseline characteristics of those providing and not providing data at each time point.

	T1		T2		Video Data	
	Teachers not providing data	Teachers providing data	Teachers not providing data	Teachers providing data	Teachers not providing data	Teachers providing data
Baseline variables	*N* = 40	*N* = 166	*N* = 78	*N* = 128	*N* = 110	*N* = 96
Age, mean (SD)	40.2 (8.5)	38.8 (9.1)	38.3 (8.6)	39.5 (9.2)	38.0 (8.5)	40.2 (9.4)
Female, n (%)	26 (65)	134 (81)	59 (76)	101 (79)	82 (75)	78 (81)
Number of years teaching, median (IQR)	14 (10, 20)	11 (6, 18)	12 (7, 18)	11 (6, 19)	12 (7, 17)	11 (6, 19)

Table S2. Comparison of teacher primary outcomes and mechanism measures at post-intervention follow-up (T2), adjusting for baseline (T0).

Outcome	mean (SD) [a]	Unadjusted mean [b] difference	Adjusted mean difference [c]				Effect size [d]	
			estimate	95% CI	p value	ICC [b]	estimate	95% CI
FFMQ – SF								
Instructor-Led 4 day	56.9 (5.3)	Ref			0.05	0.000		
Instructor-Led one-day	58.0 (6.3)	1.2	0.1	-2.7 to 2.9			0.01	-0.51 to 0.54
Self-taught 4 day	55.1 (6.7)	-1.7	-1.5	-4.5 to 1.4			-0.29	-0.85 to 0.27
Self-taught one-day	54.5 (5.7)	-2.4	-3.1	-5.6 to -0.7			-0.59	-1.04 to -0.13

[a] Raw data

[b] From model including training group variable only

[c] From model including training group variable, baseline levels of outcome and other baseline variables – gender, age, cluster size (<6 v. ≥6)

[d] Effect size (Glass's Δ) calculated as adjusted mean difference divided by the standard deviation in the instructor-led 4 day group

Table S3. Summary of costs used in economic analyses.

Item	Cost
Personal-mindfulness course materials (self-taught)	£10
Personal -mindfulness course materials (instructor-led)	£15
Personal -mindfulness course delivery (instructor-led)	£144 - £433*
Program training course delivery (one-day)	£200
Program training course delivery (four-day)	£750
Supply cover	£21 - £530*
Teachers’ travel	£13 - £187*
Teachers’ subsistence	£50 - £142*

* Cost varied for individuals depending on the number of teachers attending per school and the distance travelled to training sites; the range of costs applied is presented.

Table S4. Adjusted differences in mean costs and effects (proportion of teachers reaching the minimum competency threshold) among those who were randomised (primary analysis) and those who completed study protocol (scenario analysis).

Training group	Adj. difference in costs (£)* Estimate (SE)	Adj. difference in proportion of competent teachers (%)* Estimate (SE)	Incremental Cost-Effectiveness Ratio
Primary analysis (among those who were randomised)			
IL4D vs ST4D	361.21 (124.22)	-3.62 (-9.55)	135.25
IL4D vs IL1D	476.14 (98.71)	9.81 (9.36)	41.46
IL4D vs ST1D	729.80 (76.28)	12.15 (7.84)	59.13
Scenario analysis (among those who completed study protocol)			
IL4D vs ST4D	281.09 (72.00)	-17.75 (16.72)	-17.15
IL4D vs IL1D	723.57 (55.03)	18.13 (15.67)	33.29
IL4D vs ST1D	1008.85 (49.93)	15.91 (14.81)	56.32

IL4D: instructor-led, four-day; ST4D: self-taught, four-day; IL1D: instructor-led, one-day; ST1D: self-taught, one-day

* From model including intervention group and BL variables – gender, age, cluster size (<5)

Figure S1. Bootstrapped mean differences in costs and effects (proportion of teachers reaching the minimum competency threshold) among those who were randomised (primary analysis).

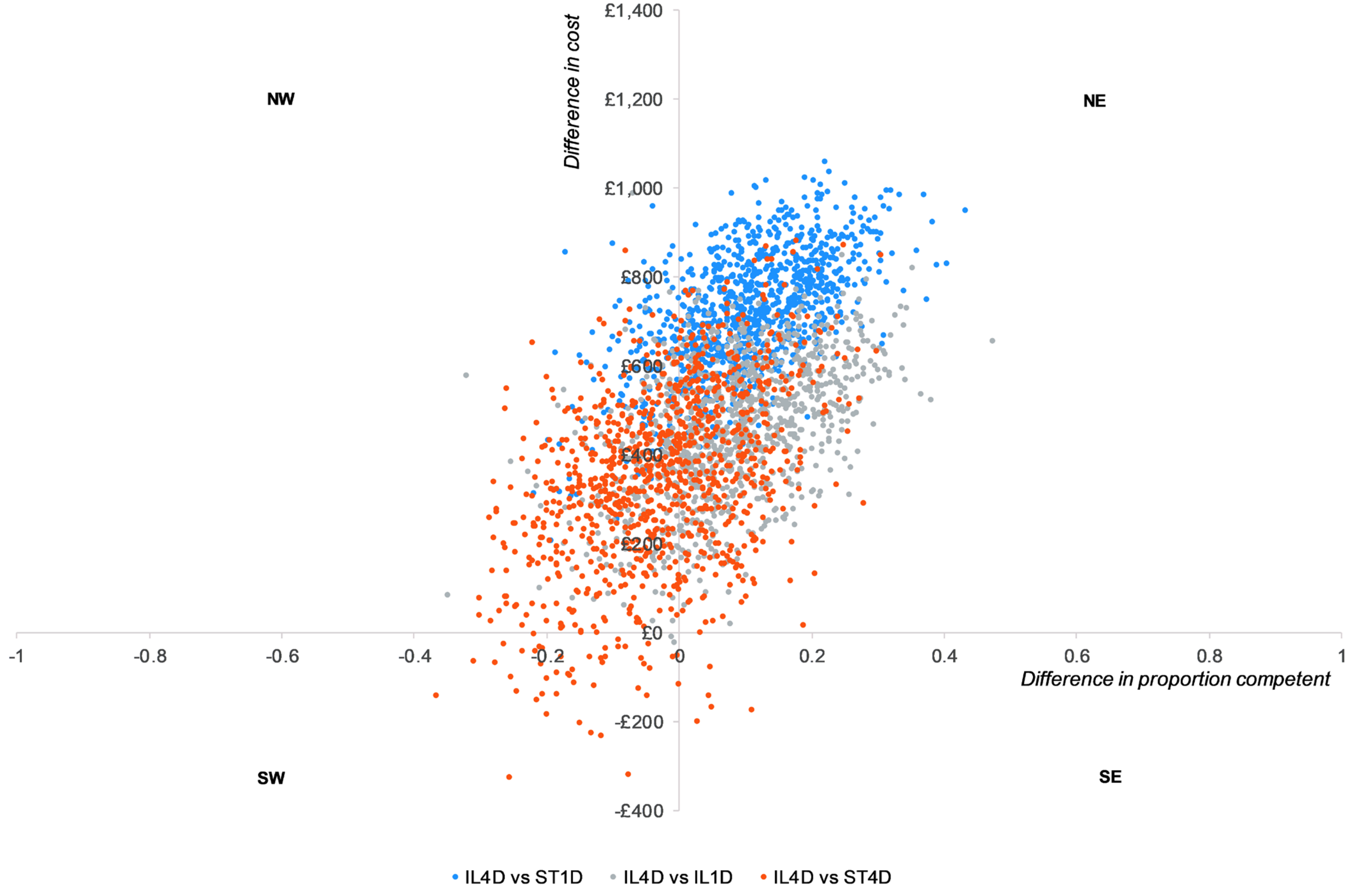

IL4D: instructor-led four-day; ST4D: self-taught four-day; IL1D: instructor-led one-day; ST1D: self-taught one-day

NW: northwest (more costly, less effective); NE: northeast (more costly, more effective); SE: southeast (less costly, more effective); SW: southwest (less costly, less effective)

Figure S2. Cost-effectiveness acceptability curve showing the probability that standard training (IL4D) is cost-effective compared to less intensive training routes for different values of willingness to pay for percentage point increase in teachers reaching the minimum competency threshold among those who were randomised (primary analysis).

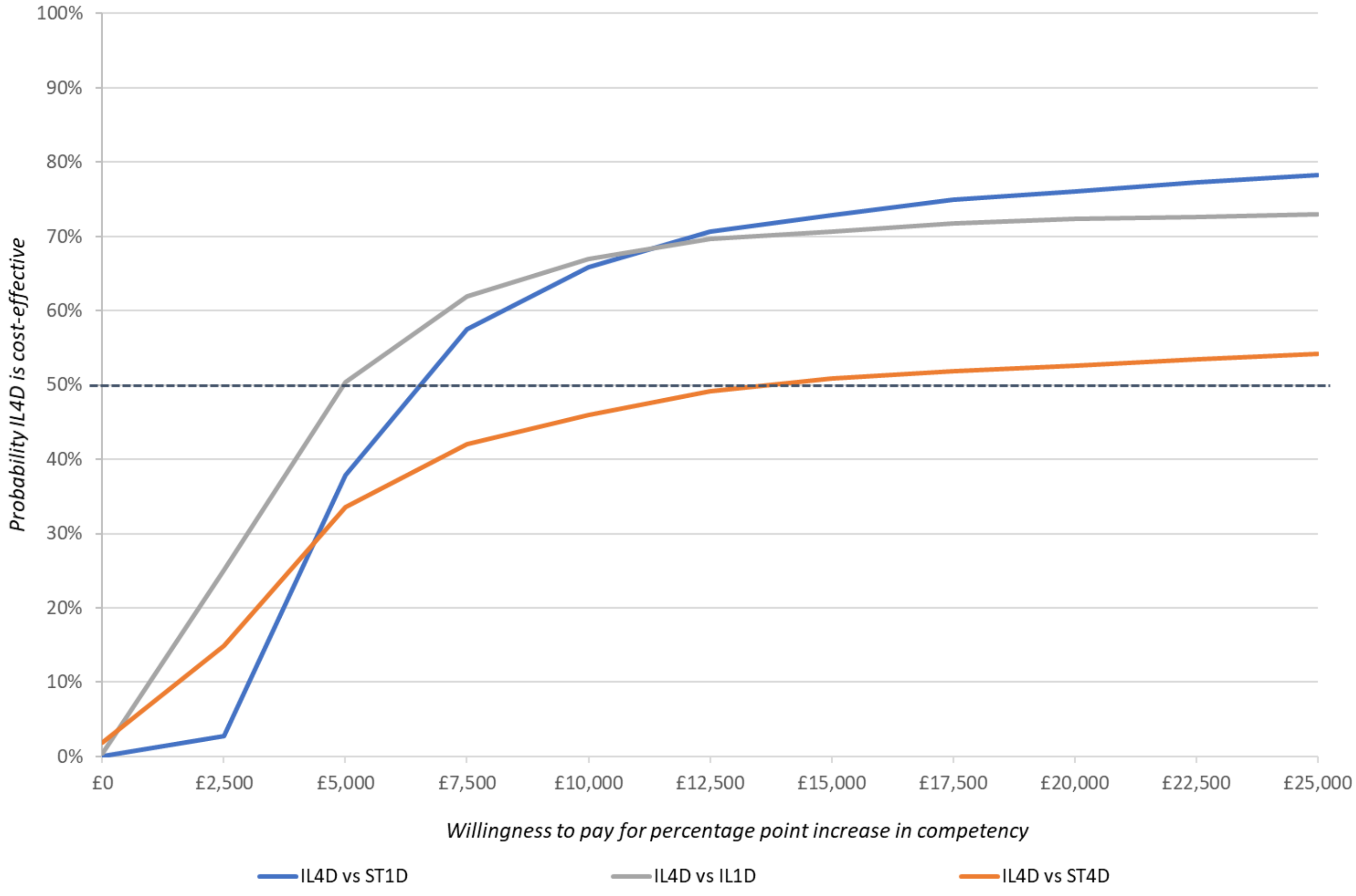

IL4D: instructor-led four-day; ST4D: self-taught four-day; IL1D: instructor-led one-day; ST1D: self-taught one-day

Figure S3. Bootstrapped mean differences in costs and effects (proportion of teachers reaching the minimum competency threshold) among those who completed study protocol (scenario analysis).

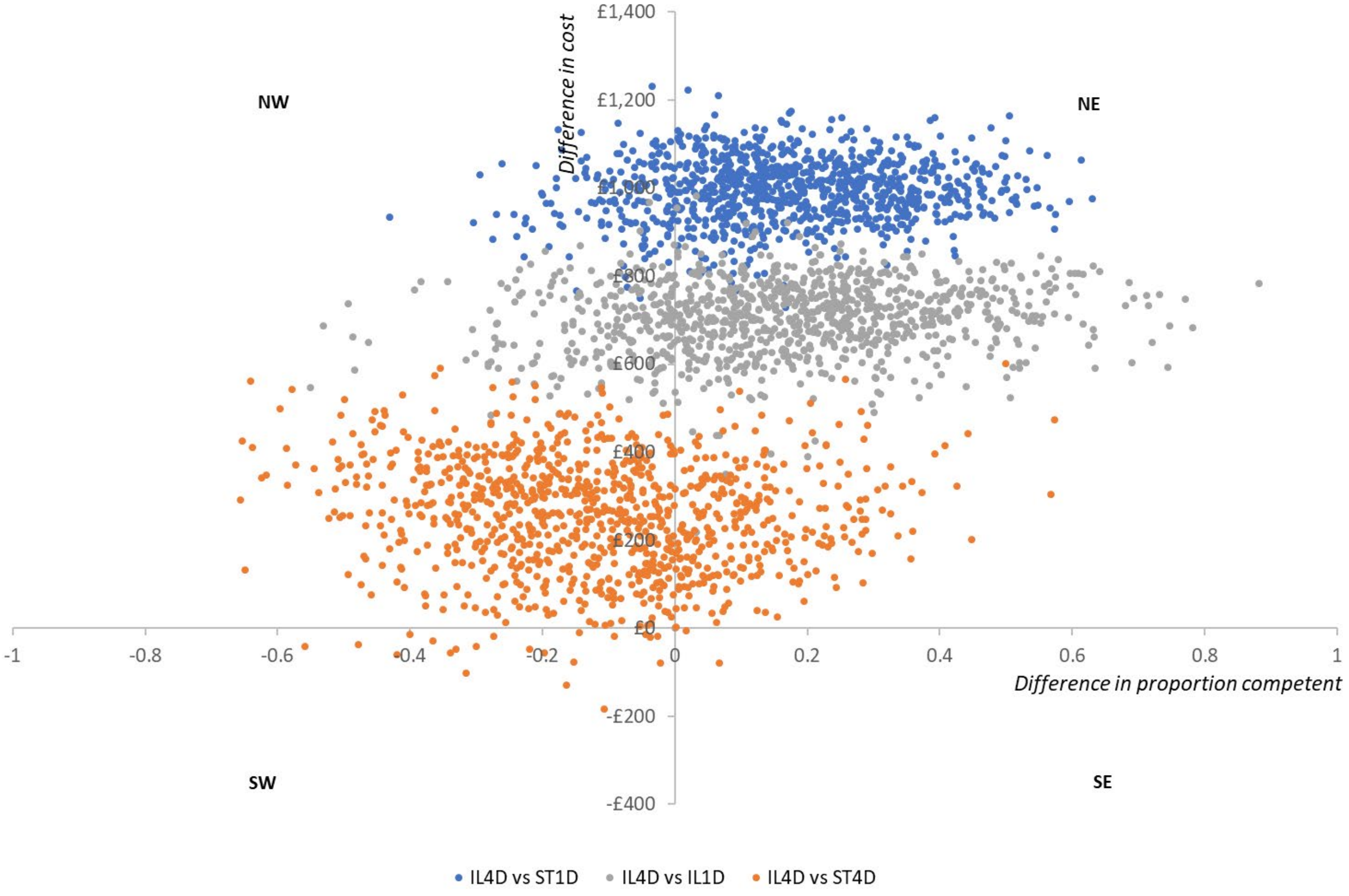

IL4D: instructor-led four-day; ST4D: self-taught four-day; IL1D: instructor-led one-day; ST1D: self-taught one-day

NW: northwest (more costly, less effective); NE: northeast (more costly, more effective); SE: southeast (less costly, more effective); SW: southwest (less costly, less effective)

Figure S4. Cost-effectiveness acceptability curve showing the probability that standard training (IL4D) is cost-effective compared to less intensive training routes for different values of willingness to pay for percentage point increase in teachers reaching the minimum competency threshold among those who completed study protocol (scenario analysis).

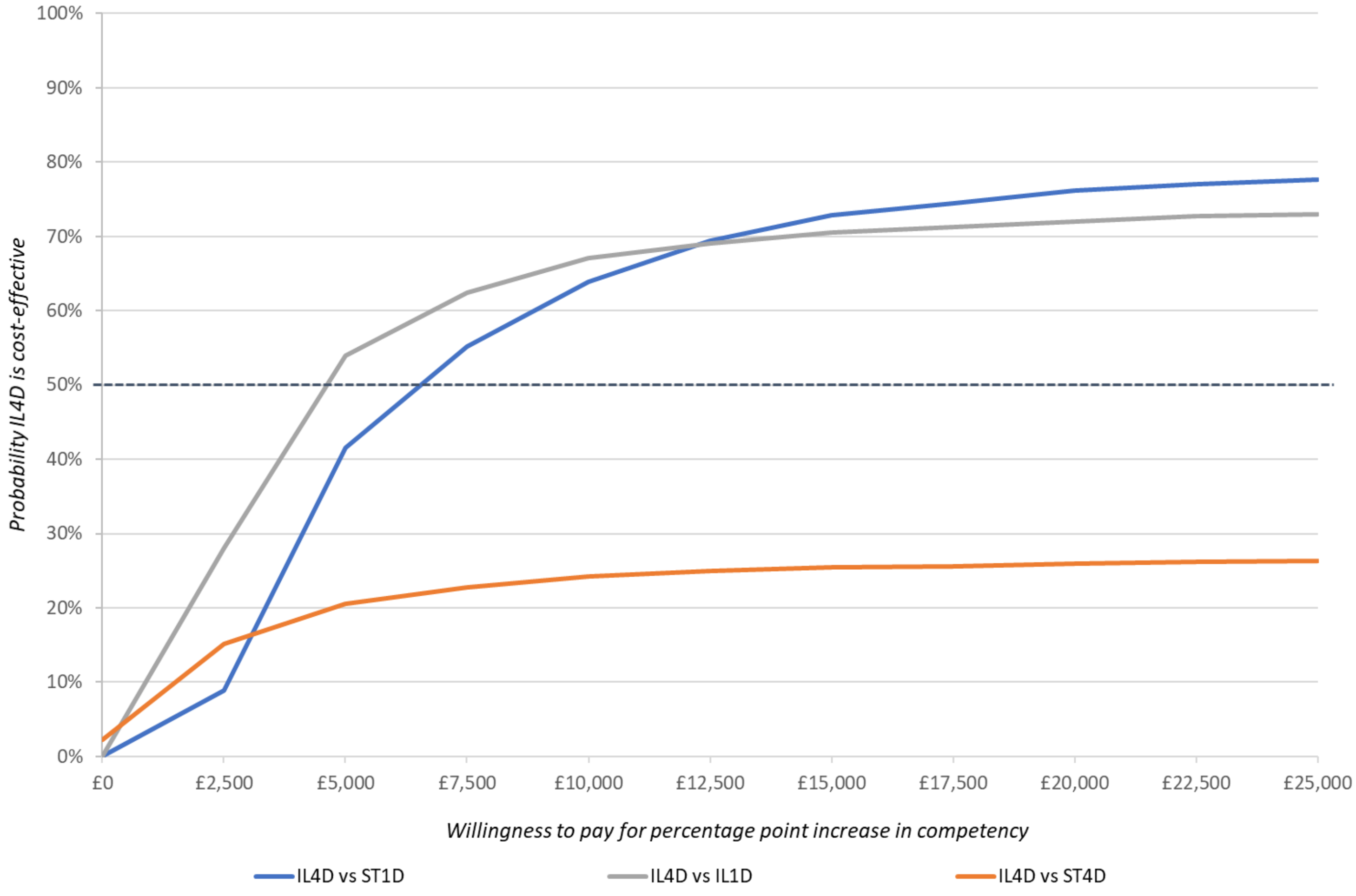

IL4D: instructor-led four-day; ST4D: self-taught four-day; IL1D: instructor-led one-day; ST1D: self-taught one-day
